# Supplementary material for: DMRforPairs: identifying Differentially Methylated Regions between unique samples using array based methylation profiles
Source: BMC Bioinformatics. 2014 May 15;15:141. doi: 10.1186/1471-2105-15-141 (PMC4046028; doi:10.1186/1471-2105-15-141)
Supplement: Additional file 2 — DMRforPairs output for the comparison of A431-MCF7 and NA17018-NA17105. Please start from the HTML files in each folder. Available via the BMC Bioinformatics website. [file 1471-2105-15-141-S2.zip › 1394847754114233_MOESM2_ESM/A431_MCF7/figures/13530.pdf]

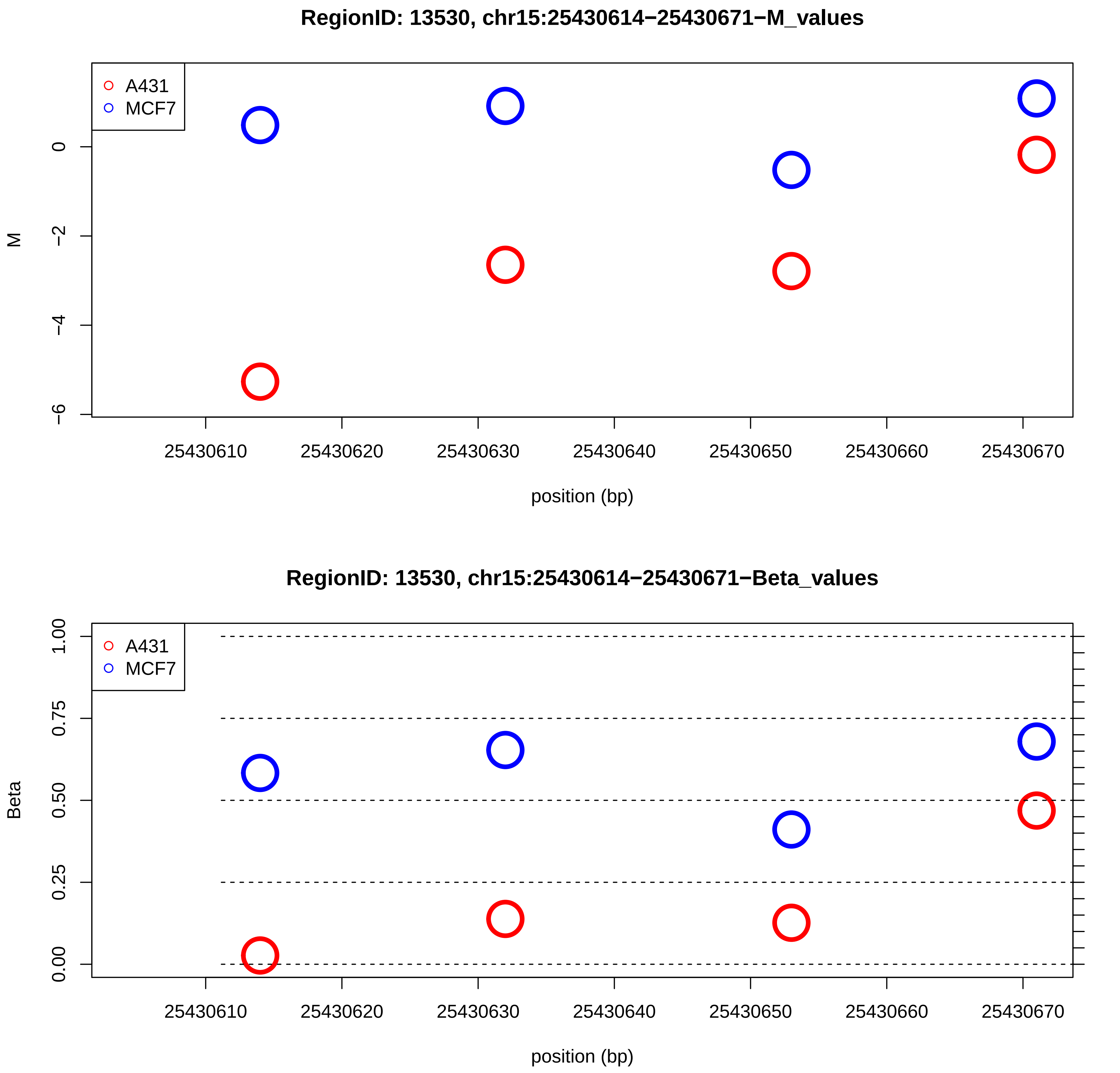

RegionID: 13530, chr15:25430614−25430671−M_values
A431
MCF7
M
0
−2
−4
−6
25430610
25430620
25430630
25430640
25430650
25430660
25430670
position (bp)
RegionID: 13530, chr15:25430614−25430671−Beta_values
A431
MCF7
Beta
1.00
0.75
0.50
0.25
0.00
25430610
25430620
25430630
25430640
25430650
25430660
25430670
position (bp)
